# Annotations

**Published:** 1903-10-10

**Authors:** 


### Annotations.

### The Overlaying of Infants.

In a recent article on this subject, Dr. Wynn Westcott reports that during the last ten years there were 15,009 overlain infants in England and Wales, and in the year 1900 there were 1,774. In London alone the yearly average of such deaths is 600, and in Liverpool 150. These figures must without doubt be considerably below the actual facts, as many cases are registered as due to "convulsions," and thus do not come under the attention of the coroner. As might be expected, the great majority of these deaths occur among the poorest classes and in the larger towns, and the immediately responsible cause is parental intemperance. To secure the punishment of a mother who has overlain her child is almost impossible. Unless there is clear evidence of culpable negligence a jury will not convict, and such evidence can rarely be obtained. The husband cannot be compelled to give evidence against his wife, and experience shows that neighbours are very reluctant to appear for the purpose of proving the woman to have gone to bed in a state of intoxication. Hence it seems useless to expect the protection of children to be secured by the infliction of punishment on the offending parent. The legislature will hardly sanction a proposal to put the burden of proof that death was accidental upon the parent when the point at issue involves a criminal charge. The radical removal of the evil is, of course, the reform of the inebriate mother—a triumph not to be achieved in a day. In the meantime some degree of protection for the children could be secured by a more general use of the cradle. In the more crowded districts of large towns the cradle is little used, partly because space is limited and partly because money is scarce. Social and temperance reformers should bear these facts in mind and should endeavour to establish a more healthy public opinion in reference to the deaths of infants by "accidental" suffocation. Coroners might co-operate in this direction by suggesting to juries that though the evidence may not be sufficient to establish a criminal charge its moral weight may justify severe censure of one or both parents.

### How to Get Rid of the Tramp.

There are two kinds of tramp. There is the man who takes to the road in search of employment, and there is the man who tramps in order that he may avoid regular work. The problem is to distinguish between the two. According to ordinary notions of justice the one should be helped to get what he wants, while the other should be, if possible, suppressed. But at present they are both treated alike, and it is perhaps partly on this account that a poor man, who would enter a hospital readily enough and accept the charity offered him there, will often endure the severest hardships rather than resort to the workhouse. Chicago is making the attempt to discriminate between the honest man who is out of work and the true vagrant. For this purpose tens of thousands of cards have been distributed bearing the legend that the municipality will give a bath, a clean and comfortable bed, a square meal and her assistance in finding employment, to every man out of work and in actual need, within her borders. In this way the tramps are attracted and placed under observation. Each is informed that he is expected to perform three hours' work on the public streets; this he does under an inspector who reports whether the manner in which the work is done indicates a real desire for employment or not. When it is clear, from the tests applied, that any man is a vagrant at heart and by choice, then the full legal penalty for vagrancy is enforced against him, but not till then. If, however, his intentions appear to be honest he will be entertained for another night or for as many nights as may be necessary. Although it may be urged, with a good display of reason, that a system of labour bureaus is all that is necessary or advisable in this country in order to help the working man who is out of employment without wasting money at the same time on the undeserving, it will be exceedingly interesting to see whether the Chicago experiment will stand the test of time.

### Temperance Teaching in the Schools.

That useful little society called the Children's Protection League deserves to be better known. It was founded to secure legal force to the clause in Viscount Peel's Minority Report to the Royal Commission on the Liquor Traffic, urging the prohibition of the sale of intoxicants to children under 16. The Intoxicating Liquors (Sale to Children) Act of 1901, as everyone knows, put the limit of age at 14, and to a large extent nullified the usefulness of the Bill by inserting the words "excepting such as are sold in corked and sealed vessels." On the passing of the Bill, the League did not dissolve, but continued for the purpose of watching and whenever possible enforcing the law, and also to suggest the introduction of elementary scientific temperance instruction in schools. Its supporters are of opinion that, good as the work of voluntary Bands of Hope is, something more far-reaching is needed, and that the time is ripe for State interference. The London School Board, it is true, does provide for suitable occasional instruction in the principles of the virtue of temperance, but the words of the code are permissive, and, further, do not apply to those children whose parents withdraw them from religious instruction. Alongside of this happy-go-lucky state of things, six public-houses are licensed to every school that is built, and then complaints are made that the national physique is deteriorating, and that the British workman is being ousted from the labour market. What the Children's Protection League asks is that the example of the United States and Canada should be followed, and scientific temperance teaching be included in the school curriculum; that the children should learn, for example, that alcohol is a poison and not a food; that it is not necessary to health or life; that its use is detrimental to vital force and power; that all the natural functions of mind and body may be performed without it; and that its habitual use predisposes the body to disease, and makes recovery from disease more difficult. And if instruction has any value at all, it should obviously, on a question of such vital importance to the health of the nation, be made definite, systematic, and compulsory

